# Long-term cardiac changes in patients with systemic lupus erythematosus

**DOI:** 10.1186/1756-0500-6-171

**Published:** 2013-05-01

**Authors:** Moacir Fernandes de Godoy, Cibele Matsuura de Oliveira, Vanessa Alves Fabri, Luiz Carlos de Abreu, Vitor E Valenti, Adilson Casemiro Pires, Rodrigo Daminello Raimundo, José Luiz Figueiredo, Glauce Rejane Leonardi Bertazzi

**Affiliations:** 1 Departamento de Cardiologia e Cirurgia Cardiovascular, Faculdade de Medicina de São José do Rio Preto, Av. Brigadeiro Faria Lima, 5416, São José do Rio Preto, SP 15090-000, Brazil; 2 Departamento de Morfologia e Fisiologia, Faculdade de Medicina do ABC, Av. Príncipe de Gales, 821, Santo André, SP, 09060-650, Brazil; 3 Programa de Pós-Graduação em Fisioterapia, Faculdade de Ciências e Tecnologia, Universidade Estadual Paulista, UNESP, Rua Roberto Simonsen, 305, Presidente Prudente, SP, 19060-900, Brazil; 4 Massachusetts General Hospital and Harvard Medical School, Building 149, 13th Street, Charlestown, MA, 02129, USA

**Keywords:** Lupus erythematosus systemic, Cardiovascular diseases

## Abstract

**Background:**

The aim of this study was evaluate the late-onset repercussions of heart alterations of patients with systemic lupus erythematosus (SLE) after a 13-year follow up.

**Methods:**

A historical prospective study was carried out involving the analysis of data from the charts of patients with a confirmed diagnosis of lupus in follow up since 1998. The 13-year evolution was systematically reviewed and tabulated to facilitate the interpretation of the data.

**Results:**

Forty-eight patient charts were analyzed. Mean patient age was 34.5 ± 10.8 years at the time of diagnosis and 41.0 ± 10.3 years at the time of the study (45 women and 3 men). Eight deaths occurred in the follow-up period (two due to heart problems). Among the alterations found on the complementary exams, 46.2% of cases demonstrated worsening at reevaluation and four patients required a heart catheterization. In these cases, coronary angioplasty was performed due to the severity of the obstructions and one case required a further catheterization, culminating in the need for surgical myocardial revascularization.

**Conclusion:**

The analysis demonstrated progressive heart impairment, with high rates of alterations on conventional complementary exams, including the need for angioplasty or revascularization surgery in four patients. These findings indicate the need for rigorous cardiac follow up in patients with systemic lupus erythematosus.

## Background

Systemic lupus erythematosus (SLE) is an inflammatory autoimmune syndrome of unknown cause that affects multiple organs, with a broad spectrum of manifestations and a clinical status marked by periods of exacerbation and remission, with a variable course and prognosis. This immunoregulatory disorder is suggested to be related with genetic, hormonal and environmental factors, resulting in chronic inflammation [1]. Antibodies act against a variety of structures, including double-helix DNA, cytoplasm antigens and antigens on the surface of cells [2]. Tissue damage may occur through the formation of immune complexes in the circulation or the presence of such sites in antigens attached to the tissues [3], involving different organs, such as the lungs, heart, kidneys, brain, peripheral nerves, skin, serous membrane and blood components. SLE occurs throughout the world, with prevalence rates ranging from 15 to 50/100,000 inhabitants. This disease predominantly affects the female gender (9:1) and the first symptoms commonly emerge between the second and third decades of life [4].

Although rare as an initial manifestation of the disease, the heart is affected in more than 50% of cases, with significant illness and mortality rates, pericarditis, myocarditis, Libman-Sacks endocarditis, pulmonary arterial hypertension and coronary disease are considered the main cardiovascular conditions associated with autoimmune alterations in SLE. In 1895, William Osler was the first to consider heart damage as part of this disease. Libman & Sacks [5] brought the world’s attention to a form of heart impairment the authors considered to be specific to Lupus, describing post-mortem findings of a form of non-infectious endocarditis, denominated typical verrucous endocarditis, in four patients with clinical data suggestive of SLE.

The frequency of heart complications in SLE is quite variable and depends on the population studied, follow-up period and methods employed in the diagnosis. Pericarditis and/or pericardial effusion are reported in 11 to 58% of cases, heart valve disease is reported in 6 to 84%, myocarditis is reported in 5 to 75%, systemic arterial hypertension is reported in 22 to 69%, heart failure is reported in 7 to 44%, ischemic coronary disease is reported in 5 to 16% and pulmonary hypertension is reported in 9 to 43% of cases [6-13].

In view of the above considerations, the objective of the study is to evaluate the evolution of patients with systemic lupus erythematosus after a 13-year follow up to analyze late-onset repercussions of heart alterations and determine the prognostic value of such an analysis.

## Method

A historical prospective study was carried out involving the analysis of data from the charts of patients with a confirmed diagnosis of SLE based on the criteria of the American College of Rheumatology treated at the Rheumatology Sector of the São Jose do Rio Preto School of Medicine (state of São Paulo, Brazil) between January 1996 and May 1997. The subjects were included in the master’s degree dissertation of one of the authors (GRLB) [7] and reevaluated in a historical prospective fashion with the aim of following the long term evolution of SLE in relation to cardiac complications. The study was approved by the Ethical committee in Research of the University in which the study was performed (Number 156/2009).

All cardiovascular exams performed since May 2009 were analyzed again for the detection of heart complications. The occurrence of cardiovascular events (angina/infarction, stroke, heart failure, arrhythmia and cardiac death) was evaluated, along with the determination of the Kaplan-Meier survival curve. For the purposes of systematization, the data was considered in three-year periods (1998 to 2000; 2001 to 2003; 2004 to 2006; and 2007 to 2009).

To analyses the late-onset repercussions, the following exams on the patients charts were used: chest x-ray, electrocardiogram, echocardiogram, cardiac stress test and coronary angiography. The need for invasive procedures for myocardial revascularization (angioplasty or surgery) was also evaluated.

All patients were followed, however, not everyone did all exams. The 26 patients that performed X-ray are not exactly the same 24 that performed ECG, which also cannot match the 10 tested for effort tests. The tests were made along the clinical follow-up, which was appointed in accordance with the need and best judgment of the clinician professionals.

## Results

Forty-eight charts of patients with a confirmed diagnosis of SLE and followed up since 1998 were analyzed, 45 (93.7%) of which belonged to female patients. Mean age was 34.5 ± 10.8 at the time of diagnosis and 41.7 ± 10.3 at the time of the analysis. Ethnic background was predominantly Caucasian (32 individuals; 66.6%), followed by Mulatto (10 individuals) and African-descent (6 individuals). Mean follow up was 7.2 ± 5.1 years (maximum: 13.6 years). Arterial hypertension was found in 30 patients (62.5%); myocardial infarction occurred in four patients (8.33%), cerebrovascular accident (stroke) was found in three patients (4.1%); and pulmonary thromboembolism occurred in one patient (2%).

### Chest x-ray

In the sequential evaluation of the four three-year periods, 26 patients were reevaluated, six of whom exhibited significant alterations: five had a normal x-ray and presented worsening and one had an altered x-ray with a worsening throughout the follow up. Seven patients had alterations on the x-ray with no worsening of the condition. Thirteen patients had normal x-rays and remained without changes throughout the follow up. Evaluation was not possible for 22 patients due to losses occurring in the last 10 years of follow up; 13 of these patients had no initial alterations and nine had initial alterations. The alterations in these patients were an increased cardiothoracic ratio, with no signs of pulmonary venocapillary hypertension.

### Electrocardiogram

In the sequential evaluation of the four three-year periods, 24 patients were reevaluated, ten of whom exhibited significant alterations: eight had a normal electrocardiogram and suffered complications and two had an altered electrocardiogram with an exacerbation of the complication throughout the follow up. Three patients had alterations with no worsening of the condition. Eleven patients had a normal electrocardiogram and remained without alterations throughout the follow up. Evaluation was not possible for 24 patients due to losses occurring in the last 10 years of follow up; 11 of these patients had initial alterations and 13 had no initial alterations. All patients exhibited sinus rhythm throughout the study.

Left ventricular (LV) overload was found in one patient in the third three-year period and two patients in the fourth three-year period. Left atrial overload was found in one patient in the fourth three-year period. Myocardial ischemia was found in one patient in the first three-year period, one in the second three-year period and two in the third three-year period; the sites of the ischemia were the inferior-apical-lateral, inferior and anteroseptal epicardium. Necrosis was found in one patient in the first three-year period, one in the second three-year period and two in the third three-year period. Altered repolarization was found in two patients in the first three-year period, five in the second three-year period, two in the third three-year period and one in the fourth three-year period. Heart arrhythmia (characterized by ventricular extrasystoles) was found in one patient in the first three-year period and two in the third three-year period. Low voltage of the QRS complex was found in one patient in the third three-year period.

### Echocardiogram

In the sequential evaluation of the four three-year periods, 26 patients were reevaluated, 12 of whom had significant changes: eight had a normal echocardiogram and suffered alterations and four had an abnormal echocardiogram with a worsening of the condition throughout the follow up. One patient had an alteration with no worsening of the condition. Thirteen patients had a normal echocardiogram and remained without alterations throughout the study. Evaluation was not possible for 22 patients due to losses occurring in the last 10 years of follow up; four of these patients had initial alterations and 18 had no initial alterations.

Pericardial effusion was detected in two patients in the first three-year period, two in the second three-year period and two in the fourth three-year period. No thickening of the pericardium was found. Pulmonary hypertension was found in one patient in the third three-year period and one in the fourth three-year period. LV hypertrophy was found in five patients in the first three-year period, two patients in the second three-year period, one patient in the third three-year period and three in the fourth three-year period. No enlargement of the left atrium or right ventricle was found, whereas enlargement of the left ventricle was found in one patient in the second three-year period.

Segmental LV hypokinesis was found in one patient in the second three-year period, one in the third three-year period and one in the fourth three-year period. Diffuse LV hypokinesis was found in one patient in the fourth three-year period. Segmental LV akinesis was found in one patient in the second three-year period and one in the fourth three-year period. LV diastolic dysfunction was found in three patients in the second three-year period and five in the fourth three-year period. LV contractile dysfunction was found in one 1 patient in the first three-year period, one in the second three-year period, one in the third three-year period and one in the fourth three-year period.

Mitral valve thickening was found in two patients in the second three-year period. Aortic valve thickening occurred in two patients in the second three-year period. Mitral valve dysfunction was found in nine patients in the first three-year period, seven in the second three-year period, four patients in the third three-year period and seven in the fourth three-year period. Aortic valve dysfunction was found in two patients in the first three-year period, two in the second three-year period and one in the fourth three-year period. Concomitant aortic and mitral valve dysfunction occurred in one patient in the first three-year period, two in the second three-year period, one in the third three-year period and two in the fourth three-year period. Mitral valve prolapsed occurred in one patient in the first three-year period and one in the second three-year period. Mitral annular calcification occurred in one patient in the second three-year period. Calcification of the posterior mitral valve leaflet occurred in one patient in the third three-year period and one in the fourth three-year period. Aortic valve calcification was found in one patient in the third three-year period and one in the fourth three-year period. Tricuspid valve dysfunction was found in two patients in the first three-year period, one in the second three-year period, one in the third three-year period and one in the fourth three-year period.

### Cardiac stress test

In the sequential evaluation of the four three-year periods, 10 patients were reevaluated, three of whom had a normal stress test and suffered significant alterations throughout the follow up. One patient had an alteration with no worsening of the condition. Six patients had a normal stress test and remained without alterations throughout the study. Evaluation was not possible for 38 patients due to losses occurring in the last 10 years of follow up; 33 of these patients had no initial alterations and five did not undergo the stress test on the initial evaluation. Results suggestive of ischemia were found in two patients in the second three-year period and one in the third three-year period. Myocardial effusion imaging revealed low uptake in the inferior wall in one patient in the third three-year period.

### Coronary angiography, transluminal percutaneous angioplasty and myocardial revascularization surgery

Coronary angiography was performed five times on four patients during the years of follow up; four cases required angioplasty and one was subsequently submitted to myocardial revascularization surgery. In the first three-year period, one patient underwent coronary angiography that demonstrated an 80% obstructive lesion in the right coronary artery (RCA), for which percutaneous transluminal coronary angioplasty (PTCA) was successfully performed. In the second three-year period, one patient underwent coronary angiography that demonstrated a 100% obstructive lesion in the RCA and an 80% obstructive lesion in the circumflex (Cx) artery, for which PTCA was successfully performed on the Cx artery, but not attempted on the RCA. In the third three-year period, two patients underwent coronary angiography One of these patients demonstrated an 80% obstructive lesion in the interventricular anterior artery (IVA), for which PTCA was successfully performed. Following a new angiography, the other case exhibited coronary disease involving the RCA and left anterior descending artery (LAD), for which myocardial revascularization surgery was performed with the saphenous vein for the RCA and the left internal thoracic artery for the LAD. In the fourth three-year period, one patient underwent coronary angiography that demonstrated a 90% obstructive lesion in the Cx artery and an 80% obstructive lesion in the diagonal branch, for which PTCA was successfully performed on both.

Table 1 summarizes the distribution of the aforementioned cardiac events based on the analysis of the complementary exams (chest x-ray, electrocardiogram, echocardiogram and stress test).

**Table 1:** Distribution of cardiac events based on complementary exams and symptoms

**Test**	**Altered with worsening**		**Altered without worsening**		**Unaltered with worsening**		**Unaltered without worsening**		**Total reevaluated**
	**n**	**%**	**n**	**%**	**n**	**%**	**n**	**%**	
**XR**	1	3.8	7	26.9	5	19.2	13	50.0	26
**ECG**	2	8.3	3	12.5	8	33.3	11	45.8	24
**ECHO**	4	15.4	1	3.8	8	30.8	13	50.0	26
**Stress**	-	-	1	10.0	3	30.0	6	60.0	10

*XR*: X-ray; *ECG*: Electrocardiogram, *ECHO*: Echocardiogram.

**Figure 1:**
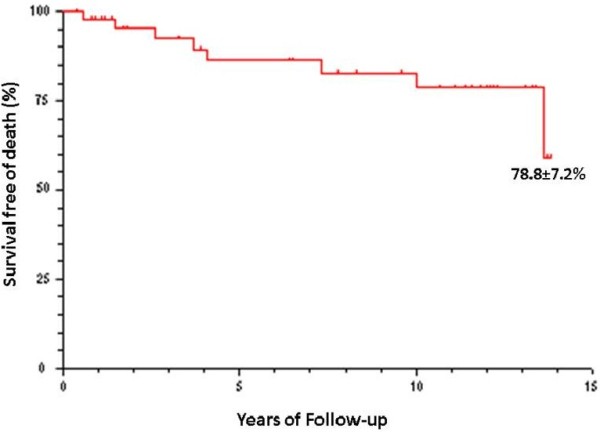
Survival curve of Kaplan-Meier for free of death at long term follow-up (until 13.6 years).

Thirty charts were active and 18 were inactive. Among the inactive charts, eight were due to the death of the patient. Two of these deaths were due to heart problems. The other causes of death were kidney disease and infectious diseases. Figure 1 displays the Kaplan-Meier graph for the percentage of death-free patients reevaluated during the 13.6 years of follow up.

## Discussion

Considering that patients with SLE are at excess risk of cardiovascular events and that the excess risk is suggested to be most pronounced in subjects under 40 years old [14], in the present study, patients with SLE and a complete cardiologic evaluation in 1998 were reevaluated through clinical exams for the diagnosis of changes in the cardiovascular system throughout the progression of the disease and its possible complications.

The genesis of the pathological mechanism involved in cardiac comorbidities in autoimmune rheumatology was enigmatic for many years [15]. Cardiomegaly was found in 50% of the patients; 11.5% exhibited LV hypertrophy and 3.8% exhibited LV dilation, which explain the enlarged heart at the time of the study. In periods of disease exacerbation, the patients may have developed myocarditis, which is difficult to detect clinically, progressing with a slight enlargement of the heart. The hyperdynamic state described by Andrade Jr. et al. [16] may also explain the enlarged heart. The increase in subepicardial adipose tissue in patients with SLE who have received corticosteroids can lead to a misinterpretation of cardiomegaly on the x-ray, as demonstrated by Bulkley & Roberts [17].

LV overload was found n 4.1% of the patients in the third three-year period of the follow up and 8.3% in the fourth three-year period. The findings are in agreement with those reported by Doherty et al. [18] in that an electrocardiogram is not sensitive enough to detect LV hypertrophy.

Myocardial ischemia was found in 4.1% of the patients in the first and second three-year periods and 8.3% in the third three-year period. Necrosis was found in 4.1% of the patients in the first and second three-year periods and 8.3% dos patients in the third three-year period. Badui et al. [7] found ischemia and necrosis in 5% and 11% of patients, respectively. Thus, these findings are similar to the study cited with regard to ischemia, but with a smaller prevalence rate regarding necrosis.

Arrhythmia was characterized by ventricular extrasystoles, which were found in 4.1% of the patients in the first three-year period and 8.3% of patients in the third three-year period. Arrythmia is generally uncommon and composed of fibrillation or atrial flutter in patients with SLE.

The prevalence rate of heart abnormalities detected by the echocardiogram was 50%, which is similar to the 54% and 57% rates reported by Nihoyannopoulos et al. [9] and Cervera et al. [10] Pericardial effusion was found in 7.6% of patients in the first, second and fourth three-year periods. This prevalence is lower than the rates described in the international literature by Leung et al. [19] (17.3%), Nihoyannopoulos et al. [9] (20.,43%) and Cervera et al. [10] (27%). However, the results of this study are similar to the prevalence rate reported by Roldan, Shively, Crawford [20] using transesophageal echocardiography (7.24%).

No cases of pericardial thickening were found, which is in agreement with Castier et al. [13] Pericardial alteration is the most frequent heart manifestation in patients with SLE. Studies report an incidence ranging from 12% to 100% when studied from the clinical standpoint [13]. Pericardial effusion was not the most prevalent cardiac abnormality in the present study, likely due to the few patients exhibiting disease activity at the time of the clinical evaluation.

Pulmonary hypertension was found in as many as 43% of patients with SLE during the clinical follow up, but is generally considered an infrequent complication with insidious development and an important cause of death. In the present study, this complication was found in 3.8% of patients in the third and fourth three-year periods.

LV hypertrophy was found in 19.2% of patients in the first three-year period, 7.6% in the second three-year period, 3.8% in the third three-year period and 11.5% in the fourth three-year period. Ong et al. [21] found LV hypertrophy in 20% of patients, which is similar to the findings of the present study. The prevalence of LV hypertrophy in patients with SLE may be explained as an alteration secondary to arterial hypertension associated with kidney failure and the effects of corticosteroids, as suggested by Doherty & Siegel [18]. In the present study, arterial hypertension was found in 62.5% of patients.

Lupus myocarditis is difficult to detect using noninvasive methods. However, in an echocardiographic study, Murai et al. [22] concluded that patients with active SLE exhibit systolic and diastolic LV dysfunction that is reversible following treatment with corticosteroids, suggesting that the heart dysfunction is subjacent to lupus activity. In the present study, diastolic LV dysfunction was found in 11.5% of patients in the second three-year period and 19.2% in the fourth three-year period. LV contractile dysfunction was found in 3.8% of patients in each of the four three-year periods. A number of etiologies have been proposed for lupus cardimyopathy, such as antibodies directed against cardiac antigens or the depositing of immune complexes, which may lead to the activation of the complement system, inflammation and myocardial damage.

In clinical practice, echocardiography has documented asymptomatic or subclinical myocardial disease, including a reduction in the ejection fraction, an increase in the heart chambers and regional hypokinesis or akinesis [12,17,22,23]. In the present study, segmental LV hypokinesis was found in 3.8% of patients in the second, third and fourth three-year periods. Global LV hypokinesis was found in 3.8% of the patients in the fourth three-year period. Segmental LV akinesis was found in 3.8% of the patients in the second and fourth three-year periods. Leung et al. [19] report an abnormality rate of 4% regarding the segmental movement of the LV wall.

The spectrum of valvulopathy related to SLE has expanded and includes valve thickening with signs of regurgitation or stenosis as well as the more characteristic valve lesion stemming from Libman-Sacks endocarditis (non-bacterial verrucous endocarditis) [11]. In the present study, endocardium-valve impairment was mainly characterized by mitral valve lesions. The most frequent abnormality was mitral regurgitation, which was found in 34.6% of patients in the first three-year period, 26.9% in the second three-year period, 15.3% in the third three-year period and 26.9% in the fourth three-year period. A number of mechanisms may be involved in the occurrence of mitral failure in patients with SLE, such as papillary muscle dysfunction, perforation of the cuspids secondary to vasculitis and fibrotic adherence of the leaflet posterior to the subjacent endocardium as a consequence of inflammation [11].

The aortic valve was affected in 7.6% of patients in the first and second three-year periods and 3.8% in the fourth three-year period, with the detection of leaflet thickening and regurgitation. Tricuspid valve regurgitation was found in 7.6% of patients in the first three-year period as well as 3.8% in the second, third and fourth three-year periods. Metz et al. [24] found aortic regurgitation in 5% of patients, tricuspid regurgitation in 5% and pulmonary regurgitation in 3%. In the present study, no pulmonary valve lesions or Libman-Sacks verrucous lesions were found. The prevalence of SLE-associated valvulopathy ranges from 18 to 74%, depending on the group studied, disease duration and method of diagnosis (autopsy, transthoracic or transesophageal echocardiography) [9,12,25,26].

Results of the stress test suggestive of ischemia were found in three patients throughout the follow up. Low uptake in the inferior wall was found in only one patient, in the third three-year period.

Myocardial infarction was found in four patients (8.33%) and stroke was found in three patients (4.1%). The prevalence of myocardial infarction varies in the literature depending on the method employed. Retrospective studies considering only clinical coronary manifestations report a prevalence rate of 6%, whereas this figure ranges from 10 to 54% in prospective studies and autopsies [27].

While not yet fully clarified, atherogenesis in SLE may be related to the characteristics of the disease and its treatment. Atherosclerosis is well defined as a pathological process induced by the use of corticosteroids [16,28,29]. Complications stemming from this condition, especially cardiac events, occur more frequently in patients with SLE for more than five years [27,30,31]. Immunoglobulins and components of the complement are identified on the wall of extramural coronary arteries with vasculitis. Cytokines, especially interleukin 6, have a proliferative effect and this action on fibroblasts may contribute to the formation of atherosclerotic plaque. The deposition of immune complements may lead to arteritis, luminal narrowing and consequent tissue ischemia [11].

Manzi et al. [32] found that female patients with lupus between 35 and 44 years of age had a 52-fold greater risk of cardiovascular events than women in an age-matched control group. In contrast with the general population, three out of four patients with lupus with coronary impairment in the present study were under 55 years of age. Thus, the acceleration of atherosclerosis in patient with SLE is an undisputable problem, as demonstrated by the illness and mortality rates stemming from myocardial infarction, which is its main form of presentation.

While the survival rate among patients with SLE has increased significantly, the mortality rate continues to be high (approximately threefold greater than that found in the general population) [33]. In the present study, death occurred among eight patients. Infection was the main cause, occurring in four patients (50%), followed by cardiovascular disease (2 patients; 25%) and nephropathy (2 patients; 25%). The deaths by cardiovascular disease were due to heart failure stemming from lupus myocarditis.

Analyzing 55 patients with a diagnosis of SLE, Costallat et al. [34] found cardiovascular disorders to be the second leading cause of death (5 cases with arrhythmia and 2 cases with congestive heart failure). In 22 autopsies of patients with SLE, Paiva et al. [35] found that the pericardium was the most often involved (45.5%), followed by the myocardium (36.4%) and endocardium (22.7%). A proportional increase in death secondary to cardiovascular alterations has been observed with the increase in life expectancy of patients with SLE [36,37]. In this context, Belibu et al. [38] proposed SLE particularities, since they reported in in North-East Romania a prevalence of low disease activity and small impairment disease characteristics may be related to the effects of early individualized therapy or SLE a specific profile. On the other hand, the relationship between cardiovascular disorders and rheumatologic diseases may be observed often in different situations because rheumatologic disorders may affect the heart and vessels [39].

The present study presents some points that are worth to be raised. It was used descriptive statistical to affirm the cardiac events of patients of this research had relation to systemic lupus erythematosus disease. However, those events could be caused by the aging and atherosclerosis or other causes.

## Conclusion

The historical prospective analysis of 48 patients with a confirmed diagnosis of systemic lupus erythematosus demonstrated progressive heart impairment over a 13-year period, with high rates of abnormalities on conventional complementary exams (chest x-ray, electrocardiogram, echocardiogram and stress test), including the need for angioplasty or revascularization surgery in four patients. The survival rate at the end of 13 years was 78.8 ± 7.2%, indicating a considerable mortality rate when considering the young mean age of the sample. The findings of the present study indicate the need for rigorous cardiac follow up in patients with systemic lupus erythematosus.

### Consent

Written informed consent was obtained from the patient for publication of this report and any accompanying images.

## Abbreviations

SLE: Systemic lupus erythematosus; LV: Left ventricular; RCA: Right coronary artery; PTCA: Percutaneous transluminal coronary angioplasty; Cx: Circumflex artery; IVA: Interventricular anterior artery.

## Competing interests

The authors declare that they have no competing interests.

## Authors’ contributions

MFde G, CMde O, VAF, LCde A, VEV, ACP, RDR, JLF and GRLB authors participated in the acquisition of data and revision of the manuscript. All authors conceived of the study, determined the design, performed the statistical analysis, interpreted the data and drafted the manuscript. All authors read and gave final approval for the version submitted for publication.
